# The effect of gamified robot-enhanced training on motor performance in chronic stroke survivors

**DOI:** 10.1016/j.heliyon.2022.e11764

**Published:** 2022-11-21

**Authors:** Arzu Guneysu Ozgur, Maximilian J. Wessel, Jennifer K. Olsen, Andéol Geoffroy Cadic-Melchior, Valérie Zufferey, Wafa Johal, Giulia Dominijanni, Jean-Luc Turlan, Andreas Mühl, Barbara Bruno, Philippe Vuadens, Pierre Dillenbourg, Friedhelm C. Hummel

**Affiliations:** a Computer Human Interaction in Learning and Instruction (CHILI), École Polytechnique Fédérale de Lausanne (EPFL), Lausanne, Switzerland; b Division of Robotics, Perception and Learning (RPL), EECS, KTH Royal Institute of Technology, Stockholm, Sweden; c Defitech Chair of Clinical Neuroengineering, Neuro-X Institute (INX) and Brain Mind Institute (BMI), École Polytechnique Fédérale de Lausanne (EPFL), Geneva, Switzerland; d Defitech Chair of Clinical Neuroengineering, Clinique Romande de Réadaptation, Neuro-X Institute (INX) and Brain Mind Institute (BMI), École Polytechnique Fédérale de Lausanne (EPFL Valais), Sion, Switzerland; e University of San Diego, San Diego, CA, USA; f School of Computing and Information Systems, University of Melbourne, Victoria, Australia; g Bertarelli Foundation Chair in Translational NeuroEngineering, Center for Neuroprosthetics and School of Engineering, Swiss Federal Institute of Technology (EPFL), Lausanne, Switzerland; h Clinical Neuroscience, University of Geneva Medical School, Geneva, Switzerland; i Neurological Rehabilitation Department of Clinique Romande de Réadaptation (SUVA), Sion, Switzerland; j Department of Neurology, University Hospital and Julius-Maximilians-University, Wuerzburg, Germany

**Keywords:** Gamification, Robotics, Stroke, Motor rehabilitation, Robot-assisted training, Personalization

## Abstract

Task-specific training constitutes a core element for evidence-based rehabilitation strategies targeted at improving upper extremity activity after stroke. Its combination with additional treatment strategies and neurotechnology-based solutions could further improve patients' outcomes. Here, we studied the effect of gamified robot-assisted upper limb motor training on motor performance, skill learning, and transfer with respect to a non-gamified control condition with a group of chronic stroke survivors. The results suggest that a gamified training strategy results in more controlled motor performance during the training phase, which is characterized by a higher accuracy (lower deviance), higher smoothness (lower jerk), but slower speed. The responder analyses indicated that mildly impaired patients benefited most from the gamification approach. In conclusion, gamified robot-assisted motor training, which is personalized to the individual capabilities of a patient, constitutes a promising investigational strategy for further improving motor performance after a stroke.

## 1 Introduction

Stroke is a major contributor to the global burden of disease [1]. It has been estimated that the absolute number of stroke survivors remaining disabled after an ischemic stroke has increased 1.4- to 1.8-folds between 1990 and 2013 [1]. Particularly, many stroke survivors are affected by upper limb motor impairment, in which magnitude largely determines the successful reintegration into an independent personal and professional life [2, 3]. This current state urgently requires further development of efficient treatments for post-stroke motor rehabilitation.

Currently, task-specific training constitutes a core element of evidence-based upper extremity rehabilitation programs [4]. Task-specific training relies on the finding that repetitive and consistent practice of meaningful and challenging tasks optimally engage intrinsic neuronal plasticity and can result in meaningful functional improvements [5]. Task-specific training can be incorporated or combined with other emerging behavioral interventions such as constraint-induced movement therapy, mirror therapy, motor imagery/mental practice [6] and neurotechnology-based solutions such as robot-assisted training [4, 7, 8]. Recently, a set of research strategies has been proposed aiming to further facilitate the design of efficient novel rehabilitation approaches and their clinical implementation [3]. Some of them are: (i) the combined application, (ii) personalization, and (iii) intensified training.

In our present work, we investigated the combined application of a gamified training strategy and robot-assisted upper extremity training. Gamification is defined as the use of game design elements in traditionally non-game contexts [9]. Common game design elements are, for instance, specific tasks (e.g., collect a target), rules (e.g., do not crash into the walls of a maze), or point systems (e.g., number of collected targets) [10]. Researchers have proposed that when designing gamified applications for rehabilitation, two game design principles are of particular importance – meaningful play and challenge [11]. Meaningful play corresponds to the presence of an apparent relationship between own actions and the system outcome for the user [12]. Challenge refers to the optimal adaptation of the task demands to the user's ability accounting for the trade-off of being potentially too easy and thereby risking loss of interest and boredom or of being too difficult, which may lead to frustration and termination of the activity [11]. Both design principles were incorporated into our tested intervention. The motor training was implemented using the Cellulo robotic platform [14, 15]. The platform consists of palm-sized, graspable, haptic-enabled tangible robots, printed paper sheets on which the robots are operating and a tablet/phone or computer controlling the application [13]. These “computer-mouse-like” robots allow users to interact with printed visual elements on paper, such as walls in a maze, and can provide visual and haptic feedback. Our main aim by using the Cellulo platform was to provide an intuitive, easy-to-use and easy-to-set-up system for motor training that allows for tangible interaction with the game elements.

**Figure 1:**
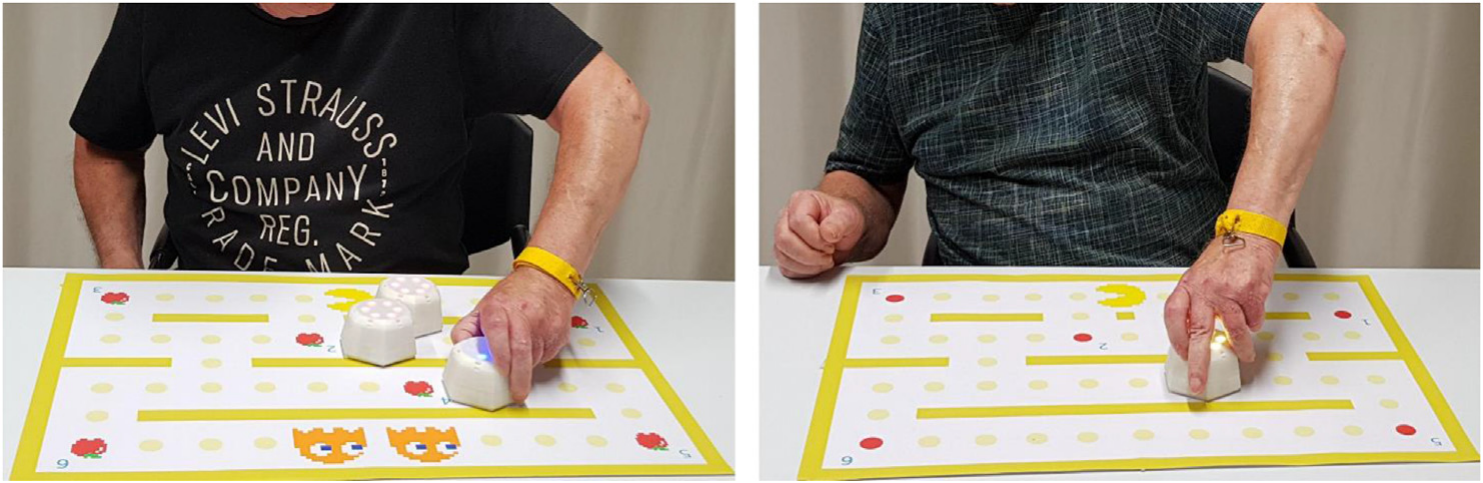
Gamified and Non-gamified training set-up. Example scenes of a patient training with gamified training modality on the left and non-gamified training modality on the right.

Previous research has tested the use of gamification strategies for rehabilitation applications, for review see, e.g., Ferreira and colleagues [10]. However, knowledge on the induced behavioral pattern and possible underlying mechanisms in clinical populations is largely lacking. In this proof-of-principle study, we strove to address this by systematically testing potential effects of a gamified robot-based upper extremity motor training in a controlled study. We hypothesized that a gamified application strategy leads to enhanced control of the robotic device during the training sessions when compared to a non-gamified control condition resulting in enhanced motor performance. Please see Figure 1 for an illustration of an exemplary scenes while a patient trains with both modalities. The respective experimental work and analyses were guided by the following research questions:

• RQ1: Does a gamified application lead to enhanced motor performance during the training phase compared to a non-gamified application?
• RQ2: Does a gamified training application lead to enhanced motor skill learning compared to a non-gamified application?
• RQ3: Does the training strategy - gamified versus non-gamified - have an impact on skill transfer to simple robot manipulation?
• RQ4: Is the magnitude of the gamification effect associated with patient characteristics determined by clinical motor scales and assessments?
• RQ5: Which training strategy do patients prefer based on their self-assessment?

In the following sections, we characterize the induced behavioral pattern and provide insights into potential underlying mechanisms of gamified robot-assisted motor training paradigms allowing one to further test the potential of haptic-enabled robot-enhanced gamified training in future randomized clinical trials.

## 2 Results

### 2.1 RQ1: Does a gamified application lead to enhanced motor performance during the training phase compared to a non-gamified application?

**Figure 2:**
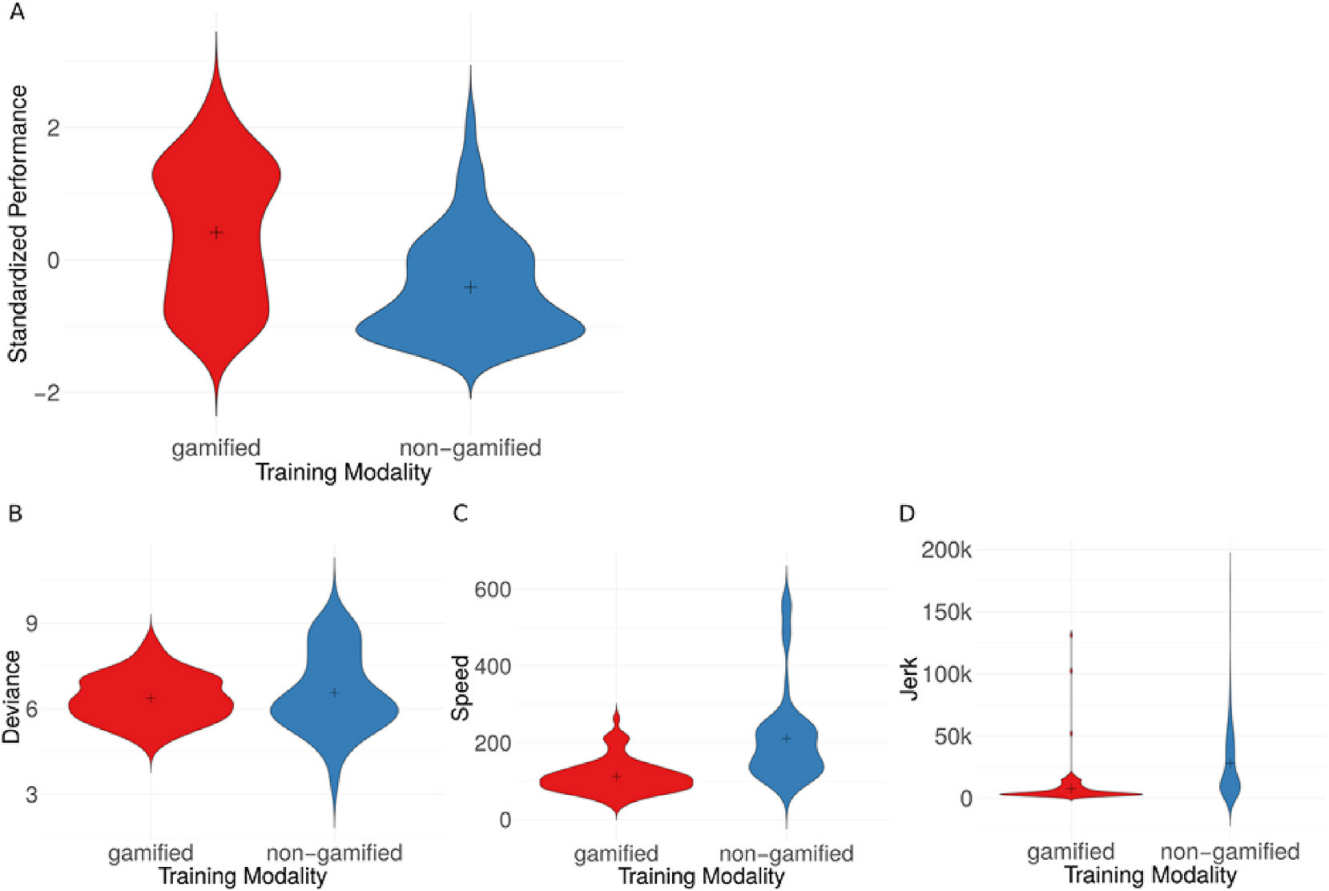
Effect of gamification on motor performance. (A) Overall motor performance is expressed via a compound performance metric defined as standardized performance = speed/(deviance ∗ jerk), normalized according to z-score, see also methods section. Cross here and in other panels indicates the mean. Higher values correspond to better performance. (B) Deviance, lower values correspond to better performance. (C) Speed, higher values correspond to better performance. (D) Jerk, lower values correspond to better performance. Depicted values of individual metrics are absolute values. Each metric is significantly higher in the non-gamified case. Deviance is in mm, jerk is in mm/s^3^, speed is in mm/s.

In order to investigate the differences in motor performance between a gamified and a non-gamified training application, we ran a hierarchical model to account for the repeated measures design. At level one, we considered the training type. At level two, we accounted for the individual participants. To control for differences in the game configurations, we included the game as a covariate in the model. We found a significant main effect of training type *t*(271) = 13.5, *p* < 0.001, *r* = 0.63, with participants having an overall higher performance during the gamified training with a large effect size (*M* = 0.41, *SD* = 1.02 for gamified training, *M* = -0.41, *SD* = 0.78 for non-gamified training) (see Figure 2A).

Next, we took a closer look at the effect of gamification on the different performance metrics, namely deviance, speed, and jerk. We ran a hierarchical model for each variable to account for the repeated measures design. At the first level, we considered the training type and at level two, we accounted for the individual participants. We included the game as covariate in the model. We found a significant main effect of training type on deviance, *t*(272) = 2.05, *p* < 0.05, *r* = 0.12, with small effect size, on speed, *t*(272) = 17.8, *p* < 0.001, r = 0.73 with large effect size, and on jerk *t*(271) = 9.4, *p* < 0.001, *r* = 0.49, with intermediate effect size. In other words, during the gamified training, participants had a smaller deviance (*M* = 6.36 mm, *SD* = 0.82 for gamified training, *M* = 6.56 mm, *SD* = 1.39 for non-gamified training), slower speed (*M* = 111.78 mm/s, *SD* = 43.69 mm/s for gamified training, *M* = 211.26 mm/s, *SD* = 117.81 mm/s for non-gamified training) and smaller jerk (*M* = 7546.61 mm/s^3^, *SD* = 14300.55 mm/s^3^ for gamified training, *M* = 27852.33 mm/s^3^, *SD* = 27306.74 mm/s^3^ for non-gamified training) (see Figure 2B-D).

### 2.1.1 Analysis of the speed accuracy tradeoff

**Figure 3:**
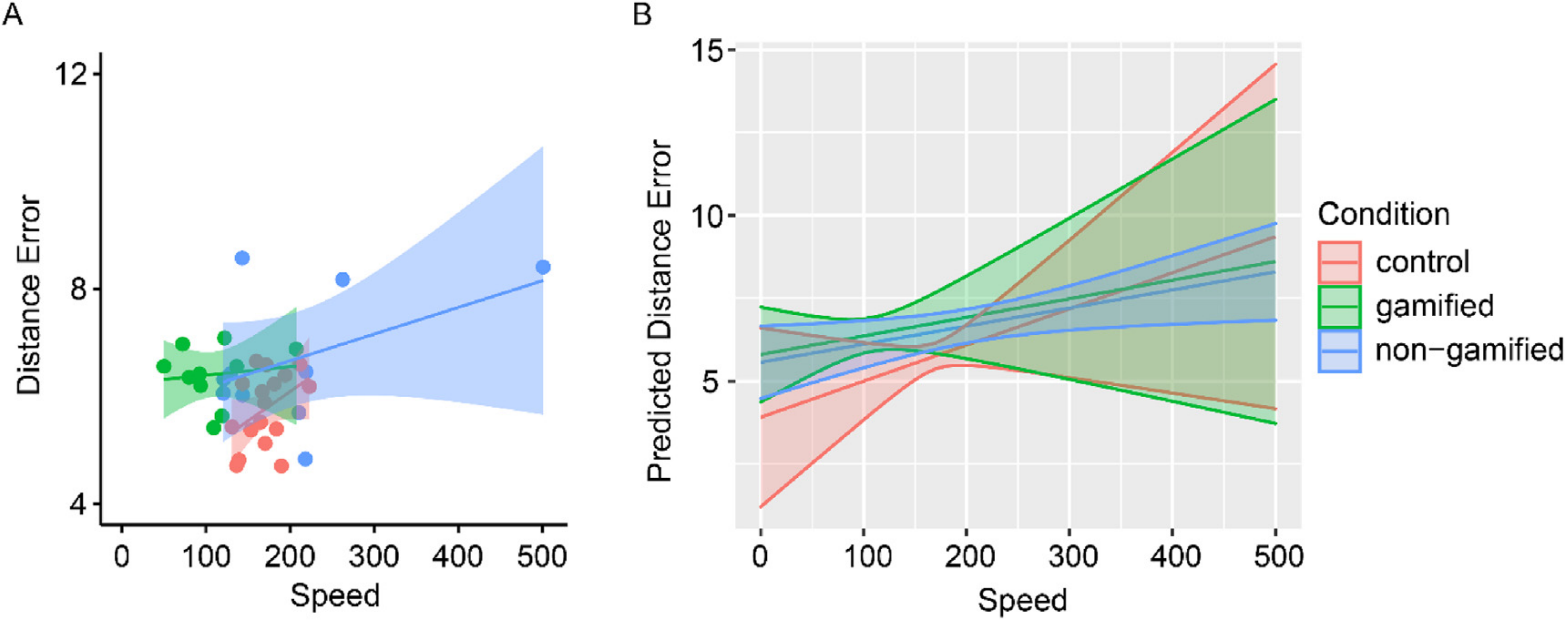
Speed accuracy tradeoff. (A) Scatterplot with linear trend lines for the independent variable speed and the dependent variable distance error (surrogate variable for accuracy) separated by experimental condition. Control cohort (red), gamified stroke cohort (green), non-gamified stroke cohort (blue). (B) Predicted regression slopes for the experimental conditions. Depicted are 95% confidence intervals.

Next, we moved on to assess whether this differential effect on the isolated performance metrics was explained by a change in the underlying speed accuracy tradeoff (SAT) across subjects. Data from a previously published age-matched control group were included in the analyses to additionally compare the observed SAT behavior with a healthy state [15]. To investigate the relationship of speed in the presence of the moderator variable experimental condition (gamified stroke cohort vs. non-gamified stroke cohort vs. gamified healthy control cohort) on the accuracy surrogate variable deviance, we ran an interaction model analysis to assess for potential differences in slopes or a shift of the linear SAT. Speed had a significant effect on the dependent variable deviance *F*(1,26.91) = 4.34, *p* = 0.047, partial eta^2^ = 0.14. The effect of the experimental condition (*F*(2,15.01) = 0.82, p = 0.46, partial eta^2^ = 0.10) or the speed × condition interaction were non-significant (*F*(2,23.29) = 0.24, *p* = 0.79, partial eta^2^ = 0.02) (Please see Figure 3A&B).

Together with the analyses of the compound and the isolated performance metrics this suggests that a gamified training strategy resulted in a more controlled motor performance characterized by a higher accuracy (smaller deviance), a higher smoothness (smaller jerk), and slower speed. Moreover, the analysis of the linear SAT suggested that the more controlled motor performance does not seem to be related to the change in its slope (non-significant interaction term) or its shift (non-significant condition term).

### 2.2 RQ2: Is a gamified training application leading to enhanced skill learning, when compared to a non-gamified application?

**Figure 4:**
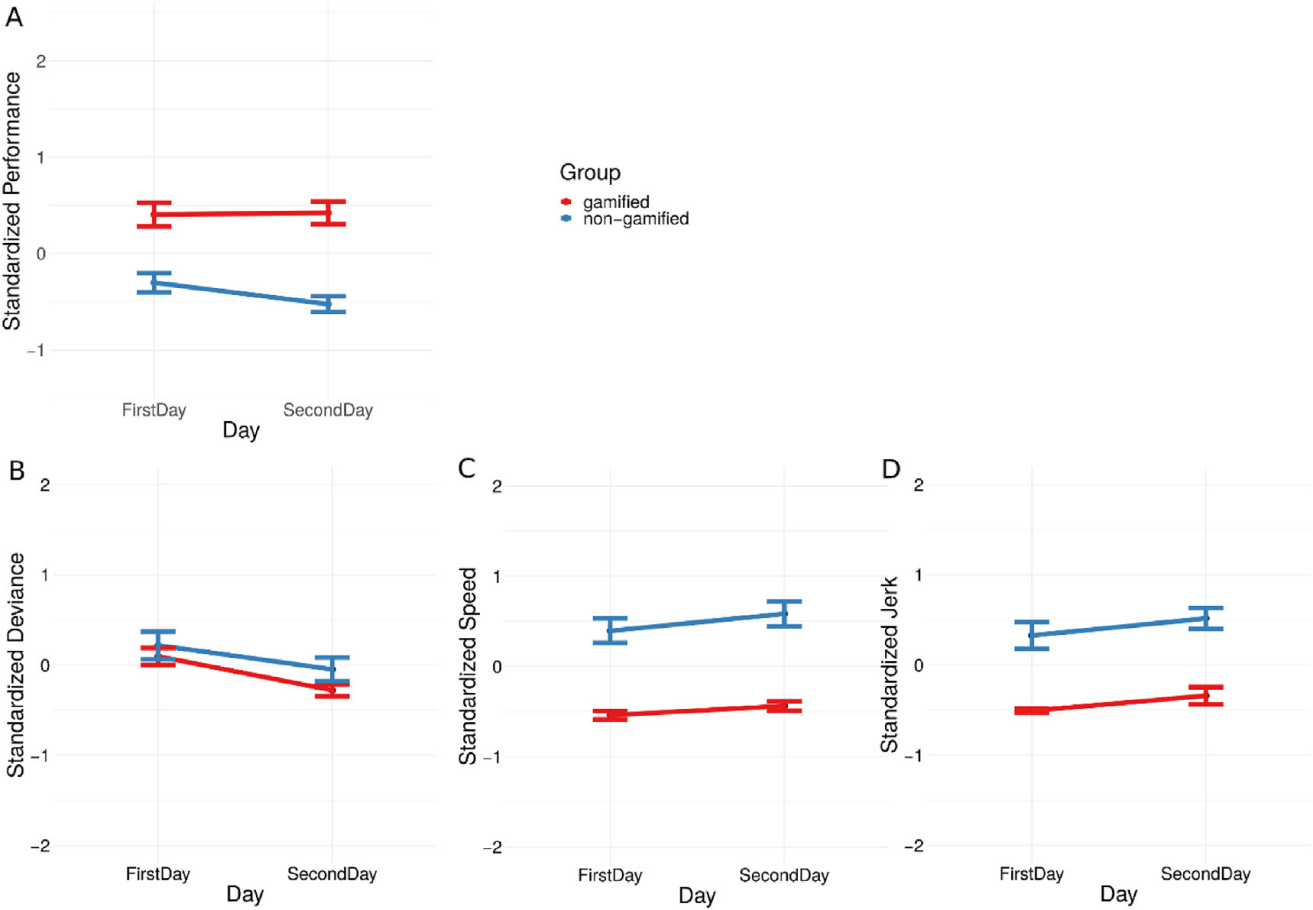
Analyses on evolution in performance across day (learning). Standardized performance separated by training type (red - gamified, blue – non-gamified). Effect of day (A) on the compound performance metric, (B) on deviance, (C) speed, and (D) jerk. Error bars indicate standard error.

To analyze if there was manifest learning from day 1 to day 2 within the participants, we conducted a hierarchical linear model to consider the repeated games for each participant. At the first level, we included day and training modality to test their effects on the standardized performance. We found a significant main effect of training type on performance, *t*(270) = 8.50, *p* < 0.001, *r* = 0.46, however, there was no significant improvement from day 1 to day 2, *t*(276) = 0.49, *p* = 0.63 and no significant interaction term between training type and day, *t*(268) = 1.52, *p* = 0.13 (see Figure 4A).

In order to evaluate for effects on the isolated performance metrics we ran a hierarchical model for each of them and accounted for the repeated measures design. At the first level, we considered the training type and day, at level two, we accounted for the individual participants and similarly we included the game as covariate in the model. We found a significant effect of day on deviance *t* 271) = 3.26, *p* < 0.01, *r* = 0.19, with participants having a smaller deviance error on the second day (*M* = 0.15, *SD* = 1.09 for the first day, *M* = -0.16, *SD* = 0.88 for the second day of the training sessions) indicating a training type-independent improvement on the isolated metric on day 2 and suggesting a learning effect (see Figure 4B). We did not find any effect of day on speed or jerk, *t*(263) = 1.2, *p* = 0.22 and *t*(277) = 0.91, *p* = 0.36, respectively (see Figure 4C-D). We found a significant effect of training type on speed *t*(271) = 12.27, *p* < 0.001, *r* = 0.66 and jerk *t*(270) = 6.56, *p* < 0.001, *r* = 0.37 respectively, but did not find a significant effect of training type on deviance *t*(270) = 1.18, *p* = 0.24.

We also did not find any interaction effect between day and gamification, for deviance, speed and jerk, *t*(269) = 0.5, *p* = 0.64, *t*(270) = 0.7, *p* = 0.48, *t*(269) = 0.1, *p* = 0.92, respectively (Please see Figure 4 B-D).

### 2.3 RQ3: Does the training strategy - gamified versus non-gamified - have an impact on skill transfer to simple robot manipulation?

In order to investigate, if there was a skill transfer for the participants in both line following activities (linear and circular line following task, for details see in the methods section below), we computed the delta performance as the difference of post-test to pre-test performance, where the positive values indicate an increase in the values from pre-test to post-test, while negative values indicate a decrease in the values for the respective contrast. We ran a hierarchical linear model to consider the repeated tests within the participant. At the first level, we considered the training type and the delta performance. At level two, we accounted for the individual participants.

**Figure 5:**
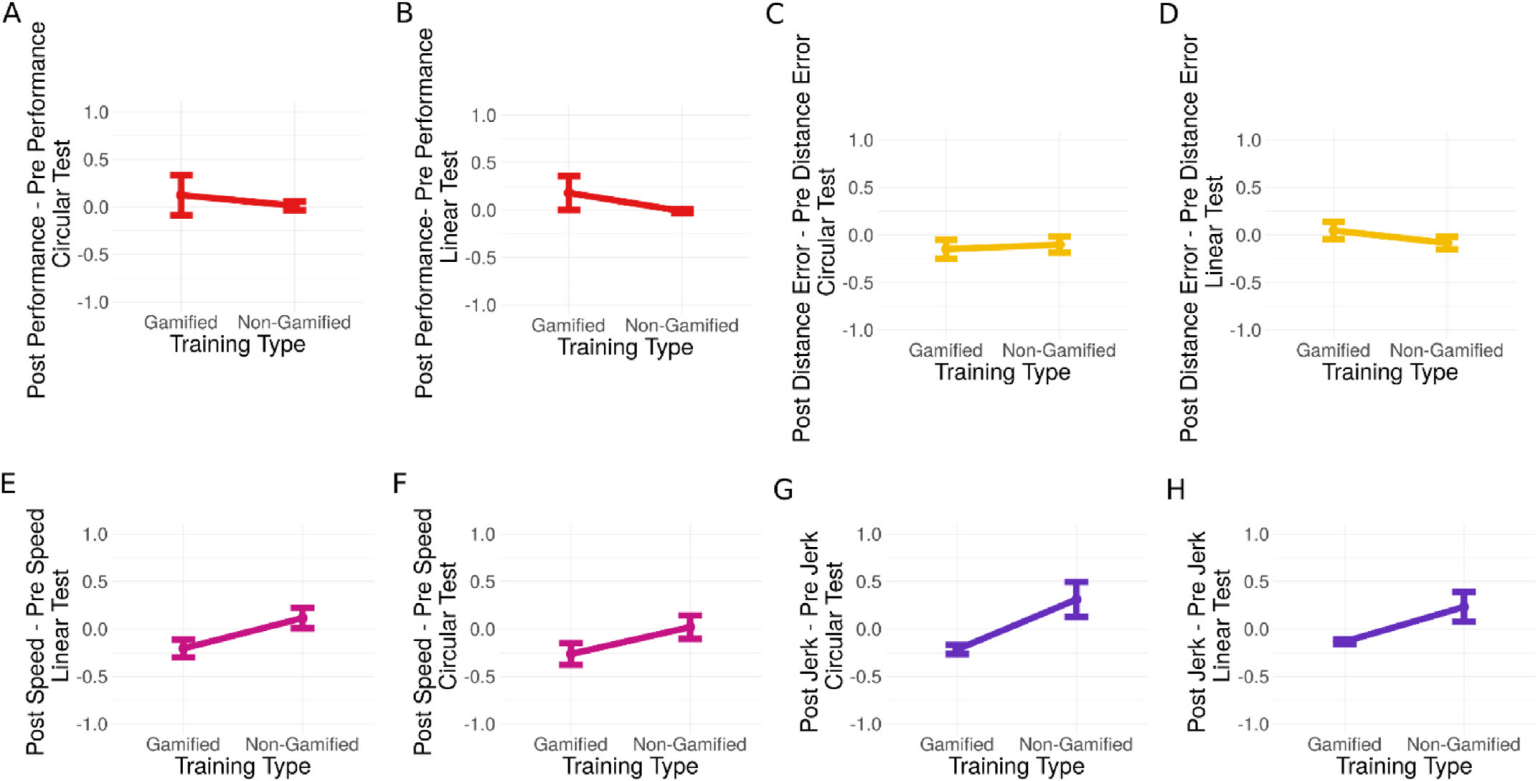
Analyses of transfer learning. Delta of compound performance metric for (A) circular line following activity and (B) linear line following activity. Error bar here and in other panels corresponds to standard error. (C, D) Distance error. (E, F) Speed. (G, H) Jerk.

For each line following tests, we applied a separate analysis. We did not find a significant main effect of training modality on the delta performance of the circular test. Similarly, we did not find a significant main effect of training modality on the delta performance of the linear test when we used the compound metric, *t*(159) = 0.50, *p* = 0.61, and *t*(245) = 1.08, *p* = 0.28, respectively (Please see Figure 5A-B).

In a follow-up analysis we investigated the effect of gamification on skill transfer for each individual metric, we ran a hierarchical model to consider the repeated tests within the participant and at the first level of each model, we considered the training type and the difference of post-test and pre-test results of the isolated performance metrics. At level two, we accounted for the individual participants.

### 2.3.1 Distance error

We did not find a significant effect of training modality on the delta distance error both for circular and linear tests, *t*(157) = 0.46, *p* = 0.64, and *t*(245) = 1.90, *p* = 0.06, respectively (see Figure 5C&D). Although there is no difference between gamified and non-gamified training modalities, we observed that in the circular test both training modalities had a negative delta (see Figure 5C), which indicates a decrease in distance error after each training modality. Upon this observation, in order to test the effect of trainings on the improvement in circular transfer activity, we run a hierarchical model to consider the repeated tests within the participant and at the first level we considered the time (pre and post) and the distance error. At level two, we accounted for the individual participants and found a significant difference between the pre-test to post-test distance error values *t*(325) = 2.66, *p* < 0.01, *r* = 0.15, where the post-tests after both trainings having lower distance error (*M* = -0.13, *SD* = 0.86) compared to the distance error of the pre-test (*M* = 0.13, *SD* = 1.11). This result suggests that in circular transfer activity, there is a training type-independent improvement on the isolated metric distance error. This is in line with the results of RQ2, where there is a training type-independent improvement in deviance from day 1 to day 2.

### 2.3.2 Speed

For the circular test, we found a significant main effect of the training type on the difference in speed from pre-test to post-test, *t*(157) = 2.44, *p* < 0.05, *r* = 0.19, where the speed increases from pre-test to post-test in non-gamified training (*M* = 0.02, *SD* = 1.12) while it decreases from pre-test to post-test in gamified training (*M* = -0.26, *SD* = 1.06) (see Figure 5E). For the linear test, we also found a significant main effect of the training type on the difference in speed from pre-test to post-test, *t*(245) = 3.07, *p* < 0.01, *r* = 0.19, where the speed increases from pre-test to post-test in non-gamified training (*M* = 0.11, *SD* = 1.22) while the speed decreases from pre-test to post-test in gamified training (*M* = -0.20, *SD* = 1.05) (see Figure 5F). These results indicate slower robot control in the post-test session for the gamified condition and the opposite for the non-gamified control condition, which is in line with the results of training performance on speed in RQ1 and might indicate a training strategy dependent transfer learning effect on the isolated performance metric speed.

### 2.3.3 Jerk

For the circular test, we did find a significant main effect of the training type on delta jerk from pre-test to post-test, *t*(157) = 3.32, *p* < 0.01, *r* = 0.26, where the jerk decreases from pre-test to post-test after gamified training while the jerk increases from pre-test to post-test after non-gamified training (see Figure 5G).

For the linear test, we also did find a significant main effect of the training type on delta jerk from pre-test to post-test, *t*(245) = 2.78, *p* < 0.01, *r* = 0.17, where the jerk decreases from pre-test to post-test in gamified training, while the jerk increases from pre-test to post-test in non-gamified training (see Figure 5H). Both analyses suggest a smoother robot control in the post-test session for the gamified condition and the opposite for the non-gamified control condition, which is indicative for a training strategy dependent transfer learning effect on the isolated performance metric jerk.

### 2.4 RQ4: Is the magnitude of the gamification effect associated with patient characteristics determined by clinical motor scales and assessments?

To assess for clinical characteristics that may determine inter-subject response variability for the gamified robot-assisted motor training intervention, the stroke cohort was split in a mildly and moderately affected subgroup based on a median split (cut-off/median: 51) of the Fugl-Meyer Assessment score data for upper extremity (FMA-UE). In a first step, we ran a hierarchical model to account for the repeated measures design. At the first level, we considered the training type and the impairment level, at level two, we accounted for the individual participants. We found a significant effect of impairment level on training modality *t*(8) = 4.01, *p* < 0.01, *r* = 0.81, indicating that stroke patients with a mild level of impairment performed better (*M* = 0.51, *SD* = 0.92), with respect to the patients with moderate level of impairment (*M* = −0.63, *SD* = 0.69).

**Figure 6:**
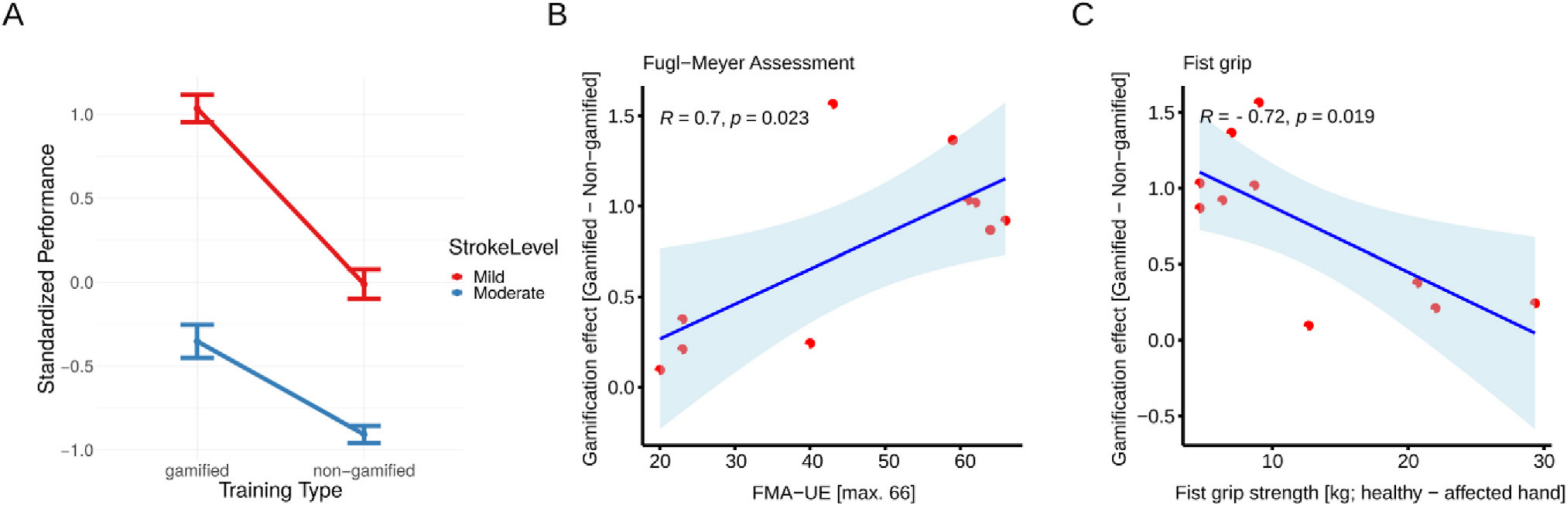
Level of motor impairment determines inter-subject response variability. (A.) Motor performance averaged across games and days for the gamified and control (non-gamifed) training intervention. Two subgroups - mild (red) and moderate (blue) motor impairment - were separated using a median split of the FMA-UA data. (B.) Pearson's product-moment correlations for FMA-UE (C.) and difference in fist grip strength, when contrasting the non-affected with the affected side, on the dependent variable gamification effect. Depicted are 95% confidence intervals.

We also found a significant interaction effect *t*(270) = 3.94, *p* < 0.001, *r* = 0.23, where the amount of difference between the performances of gamified training and non-gamified training was higher in the stroke patients having a mild level of impairment (*M* = 1.03, *SD* = 0.73, for gamified training performance, *M* = −0.01, *SD* = 0.78, for non-gamified training performance) compared to stroke patients having a moderate level of impairment (*M* = −0.35, *SD* = 0.79, for gamified training performance, *M* = −0.91, *SD* = 0.42, for non-gamified training performance). Please see also Figure 6A. Furthermore, the complementary correlation analyses indicated a positive association (*r*(8) = 0.70, *p* = 0.023) between a lower impairment level (higher FMA-UE score) and stronger gamification effect (larger individual difference to non-gamified control condition) (see Figure 6B). Additionally, the individual difference in fist grip strength, when contrasting the non-affected with the affected side, was inversely associated (*r*(8) = -0.72, *p* = 0.019) with the gamification effect (Figure 6C). These combined analyses suggest that patients with a lower motor impairment and preserved hand grip strength benefited most from the gamified training strategy.

### 2.5 RQ5: Which training strategy do patients prefer based on their self-assessment?

At the end of the experiments, the stroke survivors were asked which training strategy they preferred based on their self-assessment. The participants tended to prefer the gamified training (*N* = 6) with respect to the non-gamified training condition (*N* = 2) or prefer using both modalities according to their fatigue or level of profession in the game (*N* = 2) (*X*^2^ (2) = 4.8, *p* = 0.09).

## 3 Discussion

### 3.1 Effect of gamification on motor performance

Our main finding was that a gamified training strategy led to an overall better motor performance of stroke survivors during the training phase in comparison to a non-gamified control condition. The task execution was more controlled and characterized by more accurate (lower deviance from the optimal path), smoother (lower jerk), but slower (lower speed) movements. This result complements existing work on gamification in stroke [10, 16], by providing detailed insights on the induced behavioral pattern. Furthermore, the finding suggests that gamification approaches could be used to tune movement execution during motor practice in stroke survivors targeting the upper limb.

What are potential mechanisms that may have contributed to the induced behavioral pattern? One possibility is that this pattern was triggered by the presence of a specific set of rules and their evolution across successive games. For instance, the “wall crash penalty rule” may have encouraged the participants to favor accuracy over speed to avoid losing prior collected fruits. Furthermore, the incrementing number of rules and the accompanying increase in training complexity could have influenced the task execution speed. For example, increasing the number of chasing robots could have confronted patients with a greater number of choices they had to make during the game. This may have led to an increase in choice reaction time, via a Hick-Hyman's law-like mechanism, and thus to a slowdown of global game speed [17]. A further question was if deliberate rule violations, e.g., intentionally violating the maze boundaries, could partially drive the observed effect. Yet, the behavioral signature of rule violations is characterized by slower initiated and executed movements with a contorted trajectory [18]. In other words, a pattern, which does not match the behavioral signature detected in the present study.

Reward and punishment likely further have contributed to the gamification effect. The robotic platform incorporates reward communication capabilities by design, e.g., enchanting auditory signals, when successfully collecting a fruit. Prior behavioral work has shown that similar to the here detected behavioral pattern, reward can improve the execution of reaching movements via increasing their speed while maintaining the accuracy [19]. Conversely, punishment in our case, e.g., losing a prior collected fruit when caught by a ghost, has also shown to interact with speed and accuracy measures in motor tasks [20]. Furthermore, it seems possible that mental fatigue may have contributed to the behavioral signature of gamification, especially with increasing exercise duration, as it has been linked with movement-slowing effects in prior work [21].

In addition, disease-related features seem to determine decisions on speed accuracy relationships. For the here studied cohort, the literature suggests that stroke patients tend to choose a slower preferred movement execution speed in relation to control subjects [22]. This pattern seems to be consistent across different activities such as performing reaching movements [23] or walking [24]. The here presented analyses further addressed these research questions by studying the linear SAT across the participating stroke survivors and by benchmarking their decision tendency also to a prior acquired cohort of age-matched control participants [15]. The results indicated that the applied training strategy gamified versus non-gamified practice did not shift or change the slope of the linear SAT across subjects. Thus, a substantial influence of the gamified practice on the SAT decision process can be ruled out.

These above discussed main findings lead to the interesting question of how the gamified robot-assisted training strategy and the induced modulation of speed preference could be used for further enhancing the efficiency of task-specific rehabilitation paradigms. In fact, it has been suggested that providing tailored instructions on movement execution speed could serve as a low-cost and effective approach to boost rehabilitation [25]. Yet, there is an ongoing debate on the optimal range. For instance, some work suggests that movement quality could be improved, when stroke patients perform movements faster than their preferred spontaneously chosen speed [25, 26]. Conversely, it has been argued that a higher velocity could lead to compensatory trunk movements and impede the across joint coordination [27]. Thus, gamified training interventions could extend prior research investigating interdependencies between movement execution speed and its quality in the stroke cohort and could contribute to the resolution of the aforementioned research controversy.

### 3.2 Effect of gamification on motor learning

It has merit to briefly discuss also why gamified robotic-assisted motor practice elicited no effects on the learning rate in this study. We currently speculate that more training sessions are necessary to consistently reveal learning effects of robot-assisted gamified motor training. For instance, classical randomized clinical trials studying the value of robot-assisted upper limb rehabilitation in stroke patients, which at least partially have shown positive effects on motor impairment, employed a much larger number of training sessions. For instance, the number of sessions listed in the meta-analysis of Bertani and colleagues ranged from 15 to 36 sessions [28]. However, it is important to mention that, when we additionally examined training-related effects on fine-grained isolated performance metrics, the analyses indicated that there was an overall “training-independent” improvement in the accuracy (deviance) from day one to day two, but no improvement in speed and jerk. This could be the result of differential effect size magnitudes of the group factor (gamified vs. non-gamified) on the isolated performance metrics. In other words, the group difference in speed and jerk was having a large effect size and this might have hindered us from detecting slight differences in daily progress. Conversely, the group factor had a small effect size on deviance, which conversely may have allowed to detect slight differences in motor performance improvement across days.

Although there is no statistically significant interaction between training type and day, there might be different trends of overall performance in gamified and non-gamified training from day 1 to day 2 (Please see Figure 4A). Further long term studies are needed to explore whether patients have lower, same or higher performance after long term gameplay of both modalities and to investigate whether this might be due to patients losing interest in the non-gamified training overtime while gamified training maintains the same engagement level and performance. Long term studies with and without personalized gamification approaches should be conducted to reveal the real impact of gamified robot-mediated training on engagement and performance level by taking into account the novelty effect as well as familiarization effect [48].

### 3.3 Effect of gamification on skill transfer

To determine whether the acquired skills during the gamified and non-gamified training have any impact on daily life, we used two simple robotic manipulation activities (the circular and linear line tracing tasks (pre-/post-test), which resemble daily life activities such as using a computer mouse or wiping a table. Although we did not find a significant main effect of gamification on the performance improvement, we found some significant differences between two modalities when we looked at the isolated performance metrics in line with the findings related to RQ1 and RQ2.

Specifically, we found that speed and jerk decreased from the pre-to post-test phase after the gamified training condition only, which suggests that the gamified intervention primed a smoother and slower performance in subsequent robot control activities. These support our previous findings on gamification (RQ1) resulting in more controlled task execution with smoother and slower performance and similarly provide insights on the induced behavioral pattern. Slower robot control in the post-test session for the gamified condition might indicate a training strategy dependent transfer learning effect on speed performance where the patients might learn to manipulate the robot slower and in a more controlled way during the gamified training and sustain this behavior during the transfer activity. For instance, during the gamified training patients might be getting used to more controlled task execution due to introduced game rules (e.g., loosing points upon crushing a wall) and additional active agents (ghost robots) and sustain this controlled behavior even after the training while manipulating the robot within the simple transfer activity.

Apart from the effect of gamification, we found that overall robotic training (including gamified and non-gamified trainings) results in an improved accuracy in the transfer activity, which is in agreement with the findings of RQ2, where there is an overall improvement in accuracy from day 1 to day 2. These coherent results might be the result of successful transfer to simple motor activities, indicating the translational potential of the proposed system.

### 3.4 Association of gamification effect with patient characteristics

The analyses of the inter-subject response variability for the gamified robot-assisted training intervention indicated that stroke survivors, who were less impaired in terms of handgrip strength and synergistic upper limb movements (Fugl-Meyer scale), benefited most from the gamified training strategy. Identifying features that determine inter-subject response variability for the gamified robot-assisted intervention may allow to test personalized application strategies in the future. Such personalized approaches could be key for further stabilizing effect sizes in the individual patient [3, 29]. This is of fundamental importance, especially in light of recent negative randomized clinical trials on robotic-assisted upper limb training after stroke. As the RATULS-trial, including over 700 patients [30], have failed to show beneficial effects of a robot-assisted training using the MIT-Manus robotic gym when benchmarked to usual care. Optimally tailoring the game elements to the needs of the individual patient in a gamified robot-assisted training intervention could allow to place the patient at the individual "sweet spot" of training [31] and thus critically enhance the learning rate and resulting outcome. This emphasizes personalized gamified robot-assisted training as a promising strategy, which may be further tested in future technology aided neurorehabilitation research.

### 3.5 Preferences of stroke patients

The implementation of gamification in therapy is motivated by characteristics such as motivation, and engagement while ensuring the intended therapeutic effect of the interventions. However, there exist different levels of gamification using variants of several game elements, which are important variables to consider in the design process. For example, in our proposed non-gamified training modality, we removed the active agents, the game rules and the visual/audio/haptic feedback. However, it might have a slight level of gamification since it has one instruction, which can be accounted as a single game rule as: "Please collect as many as points possible with your robot in 3 min". On the other hand, in the gamified modality, we clearly introduce more enhanced gamification through multiple game elements, such as the autonomous ghost tracking of the patient, visual and audio feedback for patients’ actions, penalty rules, haptic informative feedback and the competitive element of losing apples when caught. Although these elements might bring attractiveness, motivation, and engagement for some patients, they might also be frustrating for other patients due to their challenging nature. The inter-individual differences in game preference could be explained by different factors such as the different sensitivity to reward and punishment or mental fatigue [46, 47].

Our results of RQ5 show that 6 patients prefer the gamified modality over non-gamified, 2 prefer the non-gamified modality over gamified and 2 prefer switching between both modalities according to their fatigue level and their level of proficiency in using the platform. This leads to the questions of "Do we need gamification for every patient?" or "Do we need the same level of gamification for every patient?" and the answer is "No" because with the same reasoning some people might prefer simple solutions compared to the challenging ones. Furthermore, this preference might not always be measurable by the amount of fatigue or frustration. For instance, one patient reported high fatigue after the gamified version, but also enjoyed the feeling of being highly focused.

According to the patients' self-reported comments, the gamified training modality was preferred by a subgroup of patients, because they found the gamified training more challenging and liked feedback mechanisms, felt more involved through the need of attention towards the game, preferred avoiding repetitiveness, and enjoyed observing the robot behavior and interacting with them. Conversely, the simpler, non-gamified version was preferred because these patients found the gamified modality more challenging and thought that it needed more attention during the training, and they wanted an easier training modality while they were tired or while having less proficiency in the game. These reports could be interpreted in the context of the flow theory [32]. Accordingly, a good balance is required between the perceived challenges of a task and one's perceived abilities, and for some patients the proposed gamification approach might not have balance these two, therefore might not have created a flow state, where the person performing the activity is immersed in a feeling of energized focus, involvement, and enjoyment.

The present results highlight the importance of personalization, adaptation, as well as the importance of having a range of training configurations for robot-enhanced training interventions. Although our study included only 10 patients and several game elements that were adapted to each patient for their own training sessions according to their abilities, there is still a need for less-gamified options with no ghost robots, less demanding options with limited game rules for patients, who preferred the non-gamified version; while there is a need for more challenging game elements such as adaptive ghost behaviors and increased difficulty levels for the patients, who preferred the gamified version.

### 3.6 Limitations

A limitation of the current work is the relatively small sample size. However, we would like to point out that the study was not designed to assess the rehabilitation potential or the clinical effectiveness. Rather, it serves as a proof-of-principle study to provide a basis for subsequent more clinically oriented trials. In these, a larger, clinically more heterogeneous sample should be tested, ideally in a multi-center design with longer training periods (e.g., 10 sessions on 2 consecutive weeks).

Furthermore, we acknowledge that the selected procedure for conducting a subgroup analysis on patient-related features that determine the responsiveness to the gamification effect may affect the outcome. Here, the median split procedure was chosen based on its simplicity.

## 4 Conclusion

To conclude, gamified and robot-assisted upper extremity motor practice resulted in more controlled performance during the training phase. The induced behavioral pattern was characterized to be a more accurate, smoother, but slower task execution. Importantly, the magnitude of the induced gamification effect was associated with the FMA-UA score and the fist grip strength of the affected hand pointing towards stronger effects in less impaired patients for the currently tested robotic setup and game elements. From an engagement perspective, most of the patients preferred a gamified training modality, but some preferred a simpler task based robotic training. These findings emphasize the importance of investigation of personalized and adaptive application strategies in related future research.

## 5 Methods

### 5.1 Participants

We recruited *N* = 10 chronic stroke survivors for the study (*N* = 5 female, mean age (range): 65 (46–72) years). The inclusion criteria were: ≥ 18 years, first ever stroke (clinically), ictal event ≥ 6 months, motor deficit, normal values of Mini-Mental State Examination (> 26/30). Exclusion criteria were: unable to consent, multiple clinical apparent strokes, cerebellar stroke, other neuropsychiatric diseases, history of seizures, high degree of spasticity (modified Ashworth Scale > 2), musculoskeletal dysfunction that compromised finger movement, pregnancy, professional musicians or intense professional usage of a computer keyboard, intake of narcotic drugs, request of not being informed in case of incidental findings. The patients were characterized with the Fugl-Meyer Assessment scale for upper extremity (FMA-UE) and grip force assessments. For an overview on the patient characteristics and stroke lesion topography, please see also Table 1.

**Table 1:** Patient characteristics and lesion topography.

Number	Lesion location	Time since stroke	Gender	Age	FMA score (max. 66)
1	Middle cerebral artery infarct left (temporoparietal and insula)	6y, 11m	M	66	64
2	Internal capsular infarct right and frontal infarct left	4y, 7m	M	72	66
3	Middle cerebral artery infarct right (frontal operculum and cortical frontal)	6y, 11m	F	71	62
4	Internal capsular and lenticular infarct right	3y, 0m	M	73	43
5	Lateral pontine infarct left	17y, 5m	F	58	59
6	Middle cerebral artery infarct left	5y, 2m	M	62	61
7	Middle cerebral artery infarct right	7y, 11m	F	46	23
8	Middle cerebral artery infarct right	19y, 0m	F	68	23
9	Internal capsular infarct left	4y, 5m	M	64	40
10	Lenticular infarct right	19y, 10m	F	72	20
Mean (range) Median split	n/a	9y, 6.2m (3y, 0m - 19y, 10m)	5F	65.2 (46–72)	46.1 (20–66) 51

The study was carried out in accordance with the Declaration of Helsinki. Written informed consent was obtained from all participants. Approval was obtained from the cantonal ethics committee Vaud, Switzerland (project number 2017-00765).

### 5.2 Experimental design

**Figure 7:**
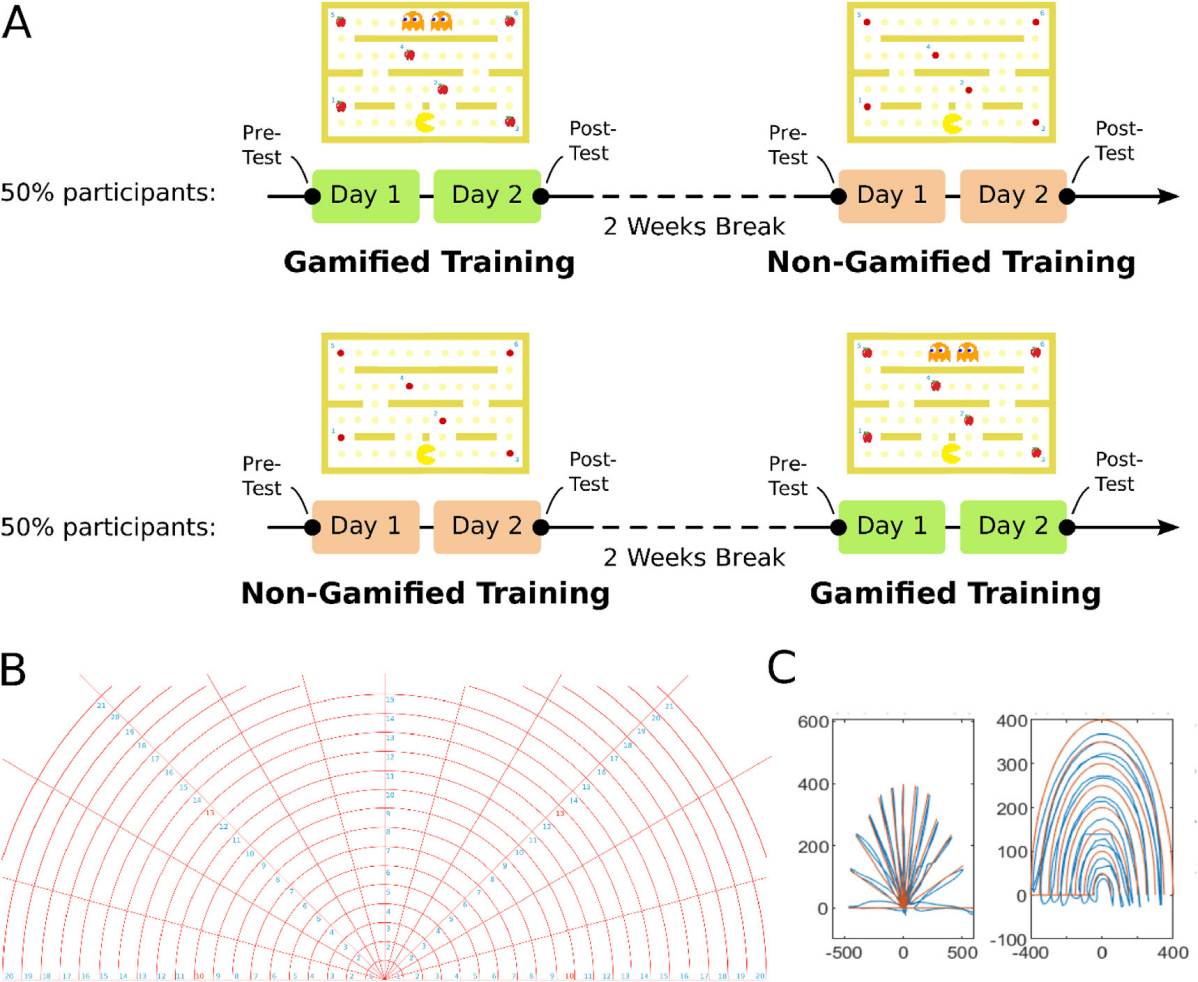
Experimental timeline and tests. (A) Experimental timeline. The study had a randomized, cross-over design. Gamified robot-assisted motor training was compared to a control condition non-gamified robot-assisted training. Each training modality was practiced on two sessions scheduled on two consecutive days. Before the interventional phase pre- and post-test assessments were conducted. (B) Pre- and post-test map to assess simple robot control via circular and linear line following activities. (C) Example performance of a stroke patient on transfer activity map with expected paths (orange-colored lines) and actual paths (blue-colored lines) generated by the patient for circular and linear tests.

The study followed a randomized, cross-over design. Each study participant practiced both training modalities, namely a gamified and non-gamified condition. Each condition was practiced on two consecutive days. A two weeks wash-out period was implemented in-between the conditions. The game map size, see also below, was customized to the patients’ needs and kept constant throughout the study for each patient. For a depiction of the experimental timeline please see also Figure 7A.

Firstly, the stroke cohort was clinically characterized using a battery of questionnaires, the FMA-UE, and grip force assessments. During the experiment, participants were sitting comfortably in front of a table with their affected hand positioned on the table to perform the training tasks. The experiment started with pre-tests consisting of circular and linear line following activities. The platform and the rules were instructed to participants for each game modality at the beginning of each game modality as follows:

**Gamified training condition:** “The goal of the game is to pick up as many apples as possible. To do so you have to move the robot in front of you with your hand. Each round lasts for 3 min. You will hear a song at the end of the 3 min, telling you that the game is over. The robots will always go back to their initial place on their own, at the beginning of each new game. The order in which you pick up the apples does not matter. When an apple is picked up, you will hear a sound and see a light appearing on your robot. You can know how many of the 6 apples you have gathered by looking at the number of lights on your robot. Once you pick the all 6 apples up, you will hear a sound and you will be able to pick up the 6 apples once again. However, you should be careful not to crash into the walls. When crashing, the robot will vibrate and will try to automatically go back to the nearest path. You cannot pick up an apple just after crashing into a wall. There will be extra robots, which are ghosts that try to catch you. In all games there is one ghost but sometimes there will be two ghosts. You have to avoid them while collecting the apples. If a ghost catches you, you will lose previously collected apples, but it is not the end of the game. After catching you, we will place it back to its starting point. You can continue to gather apples until the end of 3 min. Please perform the task as quickly and as accurately as possible. When I tell you so, you will have to rotate your ghost by 45° to pick up an apple. In some games, there will be penalties. Crashing into a wall will then make you lose one point. You then have to gather once again the last apple you picked up.”

**Non-gamified training condition:** “The goal of the game is to pick up as many red dots as possible. To do so you have to move the robot in front of you with your hand. Each round lasts for 3 min. You have to collect as much as red dots in each round as you can. Be careful to not crash into the walls. Please perform the task as quickly and as accurately as possible. In some games, when we tell you, you will have to turn the robot by 45° to collect a red dot.”

Subsequently, the participants conducted two familiarization games for the given experimental condition. Then, we started from the easiest game configuration and progressively increased the difficulty by introducing a new game element one at a time as can be seen in Table 2. On the second day, the same games were repeated. After the two days of the training session with the games, at the end of the second day the line following activities were repeated as a post-test.

**Table 2:** Game configurations per day.

Game	Day 1			Day 2		
	#Ghosts	Speed	Rules	#Ghosts	Speed	Rules
Familiarization	1	20	-			
Familiarization	1	40	T			
1	1	40	-	1	40	-
2	1	60	-	1	60	-
3	1	60	H	1	60	H
4	1	60	P	1	60	P
5	2	60	-	2	60	-
6	2	80	-	2	80	-
7	1	60	T	1	60	T
8	1	40	-	1	40	-

Each configuration consists of the number of ghosts (1 or 2), speed of the ghost(s) in mm/s (20, 40, 60 or 80), and the applied rules (H: Haptic rule, P: Penalty rule, T: Turn rule, -: No rule). Newly introduced or changed game element in blue. According to the abilities of the each patients, the configurations are adapted (e.g, decreasing the speed of the ghost by 20 mm/s or by removing the Turn rule), but the same conditions were repeated for 2 consecutive days of each modality and the total number of games played in both training modalities were kept same.

### 5.3 Robotic platform and task

Cellulo is a tabletop tangible robotic platform that works on printed paper sheets, designed to be intuitive to interact with [13]. Each Cellulo robot is small enough to be fully grasped and freely moved around by the users. In addition, each robot has six RGB-illuminated capacitive touch buttons to enable richer dynamic visual information during the interaction. The robots connect via Bluetooth to a mobile device (tablet, smartphone, laptop) that runs the activity and game logic. An optical microdot pattern on the printed sheets allows each robot to self-localize within the plane accurately (with sub-millimeter accuracy) [33]. This rich positioning data from each robot can be logged and the locomotion system within each robot is designed to be robust against rough external manipulation (typically by children and/or motor-impaired individuals) [34], while being capable of rendering haptic and tactile information [13]. With this, the platform offers a unique perspective in game design where mobile, physical game elements can be programmed to act as autonomous agents (rival, ally, neutral) as well as input devices that invoke physical exercise. To design the game logic, the printed scenery of the activity can be programmed to act as active zones with specific robot behaviors for these agents. For instance, in the tangible Pacman activity walls activate assistive haptic behavior of the robot, while fruits represent target objects to be collected in a game. Therefore, an activity is the combination of the paper sheets, the robots with particular interaction modalities and behaviors, and the mobile device that runs the activity-specific software.

### 5.4 Tangible pacman game

In order to validate the potential of Cellulo for upper extremity motor training regimes, we iteratively designed and tested a game which can be considered a tangible reimagining of the well-known Pacman arcade game [14, 50, 51]. The design was made possible by the active participation of 18 patients (stroke, brachial plexus, and cerebral palsy) and 7 therapists in 4 different therapy centers [14].

We began with a printed maze and six printed fruits in certain key intersections, where the player must move their robot (Pacman) across the maze to collect these fruits. The player is chased by active autonomous agents (ghosts) and upon a catch the player loses previously collected fruits. The player is expected to collect all six fruits as quickly and as precisely as possible (i.e., without crashing into the walls of the maze). In this version of the game, the player is given 3 min to collect as much as targets possible. Several adjustable game elements were conceived during the design process in order to dynamically adapt the difficulty of the game to the ability and training needs of each patient [14, 50, 51]:

• Maze designs and sizes for different ranges of motion (large: 980 mm × 420 mm, medium: 890 mm × 420 mm, small: 620 mm × 420 mm)
• Number of autonomous agents (ghost robots) chasing the player (1 or 2)
• Speed of ghosts chasing the player (mm/s)
• Turn rule (on/off): Fruits can only be collected by rotating the robot on top of them
• Wall crash penalty rule (on/off): Player loses last collected fruit if they crash into a wall
• Haptic feedback (on/off): Pacman provides haptic informative assistance when the player crashes into a wall.

### 5.5 Pre- and post-tests

Like other forms of real-world learning, one of the important desired learning goals of a motor training is transfer to other contexts and tasks [35, 36, 37]. In order to investigate the transfer of the learning to a simple device manipulation, we designed a line following activity as pre- and post-test including activities that are simple robot manipulation activities on defined linear and circular trajectories. Those activities are similar to holding a towel and cleaning a table-top surface as a daily activity or reaching some object on a table. The linear pre- and post-test activity includes linear trajectories with a start point at the middle bottom part of the map and the patient reaches up to a level within his/her range of motion for each line and comes back to the starting point. Each line is separated by 15° as can be seen in Figure 7B. On the other hand, the circular pre- and post-test activity includes half circle trajectories with a start point indicated by the numbers and the patient moves the robot on top of each circle starting from the affected side and comes back to the start point. Each patient performed the circular tests with the half circles that are within their range of motion. Both tests were performed on the same map in order, as can be seen in Figure 7C.

### 5.6 Data analysis

Although upper limb kinematic assessments are used in stroke research frequently, there is a lack of standardization for the use of assessment tasks, and the used kinematic metrics [38]. This presents a challenge when interpreting and comparing results. Quantitative measures of human movement quality are crucial for expressing the outcomes and clinically important changes in participants’ functional capabilities. However, the most frequently used mediums for the upper extremity functional assessment are clinical scales, which previously have been standardized and validated but have a high subjective component depending on the observer, who scores the test. Furthermore, they are not enough to assess motor performance especially during movements, and their use in combination with other more objective measures is necessary [38].

In light of the previous reviews on kinematic assessments of upper limb sensorimotor function [38, 39], the capabilities of our tangible robotic platform and the nature of our activity design, we focused on three aspects of the motion, namely accuracy, smoothness and speed. We used the following three kinematic metrics related to three motor characteristics.

• **Movement Accuracy through Mean Deviance** measures the movement quality in terms of deviation of a patient's movement from a theoretical or desired trajectory in Euclidean distance. It is considered as a measure of the error in accuracy. For the Pacman game, the optimal trajectory is estimated as the middle line of each path to account for the rectangular structure of the maze-like game. For the pre/post-tests, optimal trajectories are the provided straight lines and circular lines as in Figure 7C within the range of motion of the patient. Lower values correspond to better performance.
• **Movement Smoothness through Mean Jerk** represents the rate of change of the acceleration profile during a movement and computed from the third time derivative of position during the hand movement. It represents a measure of non-smoothness characteristic in the direction of the motion. Lower values correspond to better performance.
• **Movement Speed through Mean Speed** is the norm of the velocity of the user motion attained during the complete movement (e.g., complete game) and computed from the speed of the hand measured through the Pacman robot as the patient's end-effector. Higher values correspond to better performance.

The reviews on kinematic assessment of therapy motions provide many singular metrics but are scarce in providing a compound metric for upper limb function. In the current work, we employed a zooming-in-approach presenting an overall performance metric (see below) first, before discussing the individual metrics. This approach was chosen to allow to associate a change in outcome with a change in skill and to mitigate that it only reflects a change in the speed-accuracy trade-off [49]. The metric was defined as, with higher values corresponding to better performance:

Performance = Speed_Mean / (Deviance_Mean ∗ Jerk_Mean)

These three metrics have unequal scales and units, this is why in designing our compound metric, we opted to multiply by the metrics affecting performance positively (i.e. speed) and divide by the metrics affecting performance negatively (i.e. deviance and jerk). This metric is designed to yield an overall assessment of performance, but may hide the individual dimensions underlying performance. This is why we also focus on each of the individual metrics throughout our analysis to see the effect of our system on different characteristics of the movement. Before the analysis, the data were preprocessed through custom written Phyton code in Jupyter Notebook.

To investigate the motor performance difference between training modalities, we focused on the overall performance of the two days of the training. Overall performance for research question RQ1 was analyzed by considering all of the game performances of all patients in both days with each training modality. For research question RQ2, learning was measured by comparing the overall performance of all repeated game performances of all patients in the first day and the second day. To address RQ3, we measured the performance metric calculated by circular and linear pre/post-tests. In order to be in line with the metrics used in the main robotic activity, we considered mean speed, mean jerk and distance error through DTW distance in the pre/post-tests. We computed the distance error through the distance error between a patient's actual performed path and the expected theoretical path within this patient's range of motion in each line through a custom-written MATLAB script. We used DTW since the distance error to each curvy and straight line computed after data collection process. In order to investigate skill transfer in both transfer activities (the circular and linear pre/post-tests), we computed the performance delta by subtracting the pre-test performance from the post-test performance. Therefore, positive delta means increase in the metric from pre-test to post-test and similarly, negative delta means decrease in the metric from pre-test to post-test. For research question RQ4, to investigate the effect of gamification on motor performance of each group of stroke patients with different levels of impairment, we separated the patients into two groups according to their FMA-UE scores with a median split.

### 5.7 Statistical analysis

In order to test the effects, we used Hierarchical Linear Model (HLM)s (also known as linear mixed-effects models [40]), which were implemented in Rstudio (Version 1.0.153). To implement the HLMs, we used the linear mixed-effects models (lmer) function. Since our data was not normally distributed, we used the Kenward-Roger method for corrections, which is suggested in the literature for using the linear mixed model to analyze non-normal data distributions [41]. For the significant results, effect sizes were computed with the r metric proposed by Lenhard and colleagues [42] and it reports the following intervals for *r: .1* to *.3*: small effect; *.3* to .*5*: intermediate effect; *.5* and higher: large effect. For all figures in the results sections, error bars represent the standard error if not specified otherwise.

The SAT was analyzed by fitting an interaction model including speed and the experimental condition as fixed factors for the dependent variable distance error applying the lmer function of the lmerTest package [[43], [44]]. Random intercepts were included for subjects. Significance was evaluated by Analysis of Variance type III with Satterthwaite approximation for degrees of freedom [43, 44]. The supplementary correlation analyses were conducted using Pearson's product-moment correlations. Effects size measures were obtained as follows: Cohen reports the following intervals for *r: .1* to *.3*: small effect; *.3* to *.5*: intermediate effect; *.5* and higher: strong effect [45]. For *F*-test-based analyses we report partial eta^2^ : 0.01: small effect, 0.06: medium effect 0.14: large effect.

## Declarations

### Author contribution statement

Arzu Güneysu Özgür: Maximilian J. Wessel: Conceived and designed the experiments; Performed the experiments; Analyzed and interpreted the data; Contributed reagents, materials, analysis tools or data; Wrote the paper.

Jennifer K. Olsen: Analyzed and interpreted the data; Contributed reagents, materials, analysis tools or data.

Andéol Geoffroy Cadic-Melchior; Valérie Zufferey: Performed the experiments.

Wafa Johal: Conceived and designed the experiments.

Giulia Dominijanni: Analyzed and interpreted the data.

Jean-Luc Turlan; Andreas Mühl; Barbara Bruno; Philippe Vuadens: Contributed reagents, materials, analysis tools or data.

Pierre Dillenbourg; Friedhelm C. Hummel: Conceived and designed the experiments; Analyzed and interpreted the data; Contributed reagents, materials, analysis tools or data; Reviewed and edited the paper..

### Funding statement

Partially supported by the Swiss National Science Foundation through the National Centre of Competence in Research Robotics (NCCR).

Partially supported by #2017-205 ‘Personalized Health and Related Technologies (PHRT-205)’ of the ETH Domain to FCH, the Defitech Foundation (Strike-the-Stroke project, Morges, Switzerland) to FCH, the Wyss Center for Bio and Neuroengineering (WCP024, Genève, Switzerland).

Partially supported by the European Research Council (ERC) under the European Union’s Horizon 2020 research and innovation programme (Grant agreement No. 813713).

Partially supported by Digital Futures Research Center, Sweden through Postdoctoral Fellowship Grant.

### Data availability statement

Data will be made available on request.

### Declaration of interest’s statement

The authors declare no conflict of interest.

### Additional information

No additional information is available for this paper.
